# Neuroprotection of Transcranial Cortical and Peripheral Somatosensory Electrical Stimulation by Modulating a Common Neuronal Death Pathway in Mice with Ischemic Stroke

**DOI:** 10.3390/ijms25147546

**Published:** 2024-07-09

**Authors:** Hongju Lee, Juyeon Lee, Dahee Jung, Harim Oh, Hwakyoung Shin, Byungtae Choi

**Affiliations:** 1 Department of Korean Medical Science, School of Korean Medicine, Pusan National University, Yangsan 50612, Republic of Korea; hongju120@pusan.ac.kr (H.L.); diane676@pusan.ac.kr (J.L.); jjj262999@pusan.ac.kr (D.J.); harim5329@pusan.ac.kr (H.O.); julie@pusan.ac.kr (H.S.); 2 Graduate Training Program of Korean Medical Therapeutics for Healthy Aging, Pusan National University, Yangsan 50612, Republic of Korea

**Keywords:** ischemic stroke, transcranial alternating current stimulation, transcranial direct current stimulation, electroacupuncture, neuronal death, inflammatory cytokine, neurotrophic factor

## Abstract

Therapeutic electrical stimulation, such as transcranial cortical stimulation and peripheral somatosensory stimulation, is used to improve motor function in patients with stroke. We hypothesized that these stimulations exert neuroprotective effects during the subacute phase of ischemic stroke by regulating novel common signaling pathways. Male C57BL/6J mouse models of ischemic stroke were treated with high-definition (HD)-transcranial alternating current stimulation (tACS; 20 Hz, 89.1 A/mm^2^), HD-transcranial direct current stimulation (tDCS; intensity, 55 A/mm^2^; charge density, 66,000 C/m^2^), or electroacupuncture (EA, 2 Hz, 1 mA) in the early stages of stroke. The therapeutic effects were assessed using behavioral motor function tests. The underlying mechanisms were determined using transcriptomic and other biomedical analyses. All therapeutic electrical tools alleviated the motor dysfunction caused by ischemic stroke insults. We focused on electrically stimulating common genes involved in apoptosis and cell death using transcriptome analysis and chose 11 of the most potent targets (Trem2, S100a9, Lgals3, Tlr4, Myd88, NF-kB, STAT1, IL-6, IL-1β, TNF-α, and Iba1). Subsequent investigations revealed that electrical stimulation modulated inflammatory cytokines, including IL-1β and TNF-α, by regulating STAT1 and NF-kB activation, especially in amoeboid microglia; moreover, electrical stimulation enhanced neuronal survival by activating neurotrophic factors, including BDNF and FGF9. Therapeutic electrical stimulation applied to the transcranial cortical- or periphery-nerve level to promote functional recovery may improve neuroprotection by modulating a common neuronal death pathway and upregulating neurotrophic factors. Therefore, combining transcranial cortical and peripheral somatosensory stimulation may exert a synergistic neuroprotective effect, further enhancing the beneficial effects on motor deficits in patients with ischemic stroke.

## 1. Introduction

Stroke is the second leading cause of death worldwide, and survivors experience various chronic disabilities, with motor dysfunction being the most severe neurological disorder [1,2]. Effective treatments to restore functional impairment in patients with stroke are limited, and their efficacy often does not meet patients’ expectations [2,3]. Ischemic stroke caused by cerebral artery occlusion induces neuronal brain death owing to a lack of blood and oxygen; accordingly, available treatments typically involve thrombolytic therapy, endovascular thrombectomy (EVT), and rehabilitation [2,4]. Thus, prospective strategies following ischemic stroke should focus on potential therapeutic tools to optimize functional recovery.

Therapeutic electrical stimulation, applied at the brain level or via the somatosensory system, is widely used to aid functional rehabilitation in patients with ischemic stroke. Transcranial-alternating current stimulation (tACS) and -direct current stimulation (tDCS), two non-invasive brain stimulation tools, show potential in alleviating motor deficits in patients with stroke by modulating cortical excitability in the central nervous system [5,6,7]. These transcranial current stimulations share some characteristics with electrical stimulations; however, the electricity used to deliver the stimulation differs. Transcranial stimulations use a dependence-oscillating sinusoidal current or polarity-specific modulation to modify cortical activity. Thus, the underlying mechanisms in the brain also differ [8,9].

The National Institutes of Health recommend acupuncture, a form of peripheral somatosensory stimulation, as an adjuvant therapy for stroke owing to its positive results and lack of overt side effects [10,11]. Electroacupuncture (EA), one type of acupuncture, employs continuous electric pulses applied to specific body acupoints via an inserted needle and is a potent form of somatosensory stimulation [12]. Combining transcranial current- and peripheral nerve-stimulation improves motor function synergistically as both tools induce bi-directional activities [13,14]. Transcranial current stimulation involves N-methyl-d-aspartate (NMDA) receptors to induce cortical excitability and synaptic plasticity [15,16,17]. The NMDA receptor also contributes to the therapeutic effectiveness of EA as a potent signal factor that enhances neuronal plasticity [18].

The penumbra or peri-infarct zone is the primary target for neuroprotective therapy after an ischemic stroke because it includes regions surrounding the core of the infarct, where blood supply is partially maintained and neurons can temporarily survive [19,20]. A significant lack of cerebral blood flow following ischemic stroke induces neuronal brain death; however, neurons in the penumbra activate survival signaling pathways immediately following the injury, which continues for several days [19,20,21]. Therefore, therapies must be applied during this early phase to accelerate recovery following a stroke while carefully considering the relevant risks to avoid potentially harmful effects [7,22].

Only a few therapeutic choices are available for patients with ischemic stroke, especially during the acute stage. EVT can reverse motor deficits in some patients but there are no universally acknowledged treatments to reverse non-motor impairments [23]. This study compared the therapeutic efficacies of electrical stimulations applied to the transcranial cortical- or peripheral somatosensory-nerves in promoting motor functional recovery during the early stages of stroke. We also aimed to identify the mechanisms underlying neuroprotection from stroke insults during the subacute stage to guide more effective therapies for following stroke rehabilitation, particularly the improvement of motor function. Our findings provide a basis for the potential use of therapeutic electrical stimulation as adjuvant therapy for patients who experience substantial motor impairment following ischemic stroke.

## 2. Results

### 2.1. Effects of Therapeutic Electrical Stimulation on Motor Restoration in Ischemic Stroke

**Figure 1:**
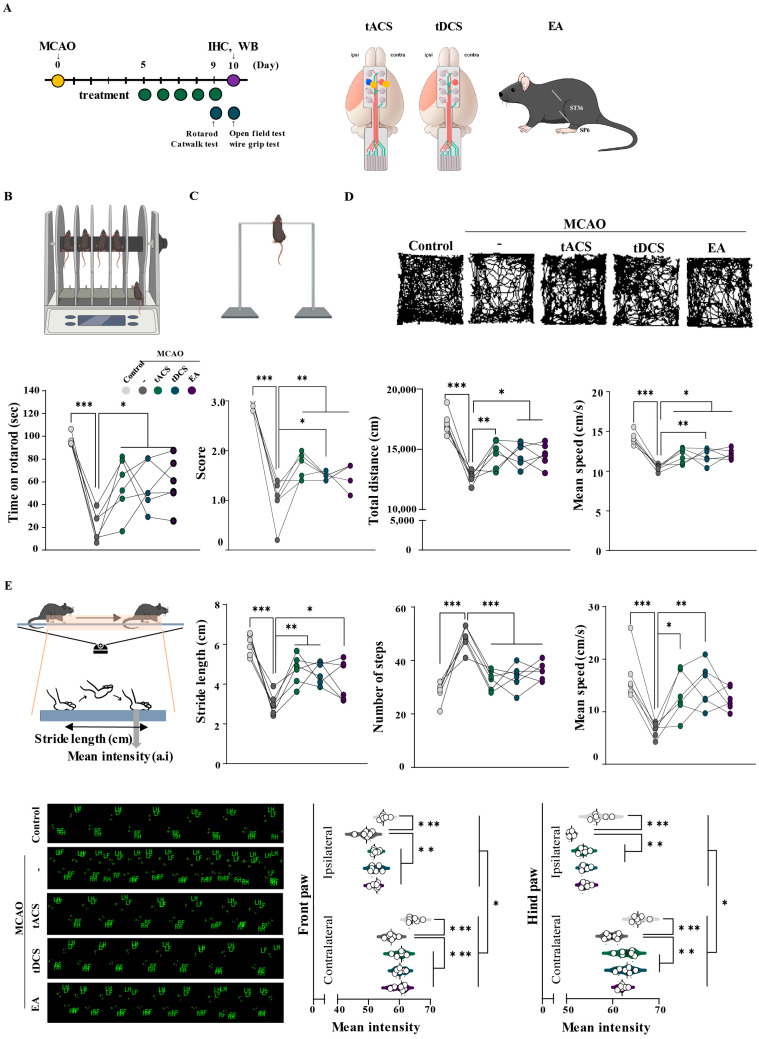
Effects of therapeutic electrical stimulation on motor restoration in ischemic stroke. (**A**) Experimental schedule and schematic images of electrical stimulation. Quantification of the results of behavioral testing, including the (**B**) Rotarod, (**C**) Wire-grip, (**D**) Open-field, and (**E**) Catwalk tests. All electrical stimulations significantly improved motor dysfunction compared with MCAO. All data are expressed as mean ± standard error of the mean (n = 6/group); * *p* < 0.05, ** *p* < 0.01, and *** *p* < 0.001 vs. each group using one-way analysis of variance with Tukey’s test. Dot color means light grey, Control; dark grey, MCAO; Green, MCAO + tACS; Blue, MCAO + tDCS; Pupple, MCAO + EA group.

Behavioral assessment was performed following electrical stimulation applied 9 and 10 days after middle cerebral artery occlusion (MCAO) to determine the therapeutic efficacy of electrical stimulations administered at the subacute stage of stroke (Figure 1A). The MCAO groups displayed significantly more intense motor dysfunction compared to the control group in all behavioral tests. In the rotarod, wire grip, and open-field tests, the electrically stimulated groups (MCAO + tACS, MCAO + tDCS, and MCAO + EA) demonstrated significant improvements in motor function compared with the MCAO-only group (rotarod test: F[4,25] = 12.796, *p* < 0.001; wire grip test: F[4,25] = 46.461, *p* < 0.001; total distance: F_[4,25]_ = 18.065, *p* < 0.001; and mean speed: F_[4,25]_ = 18.055, *p* < 0.001) (Figure 1B–D). Moreover, gait analysis in the catwalk test revealed that all treated groups demonstrated significant improvements in their ability to walk, including stride length, number of steps, mean speed, and mean intensity (stride length: F_[4,25]_ = 14.317, *p* < 0.001; number of steps: F_[4,25]_ = 23.431, *p* < 0.001; mean speed: F_[4,25]_ = 6.951, *p* < 0.001; and mean intensity: F_[4,25]_ = 13.088, *p* < 0.001), except for the mean speed and intensity of the MCAO + EA group (Figure 1E). Based on the results of these behavioral assessments, all types of electrical stimulation improved motor function in the ischemic stroke mouse model.

### 2.2. Transcriptomic Analysis of Potent Signaling Pathways in Response to Therapeutic Electrical Stimulation in Ischemic Stroke

**Figure 2:**
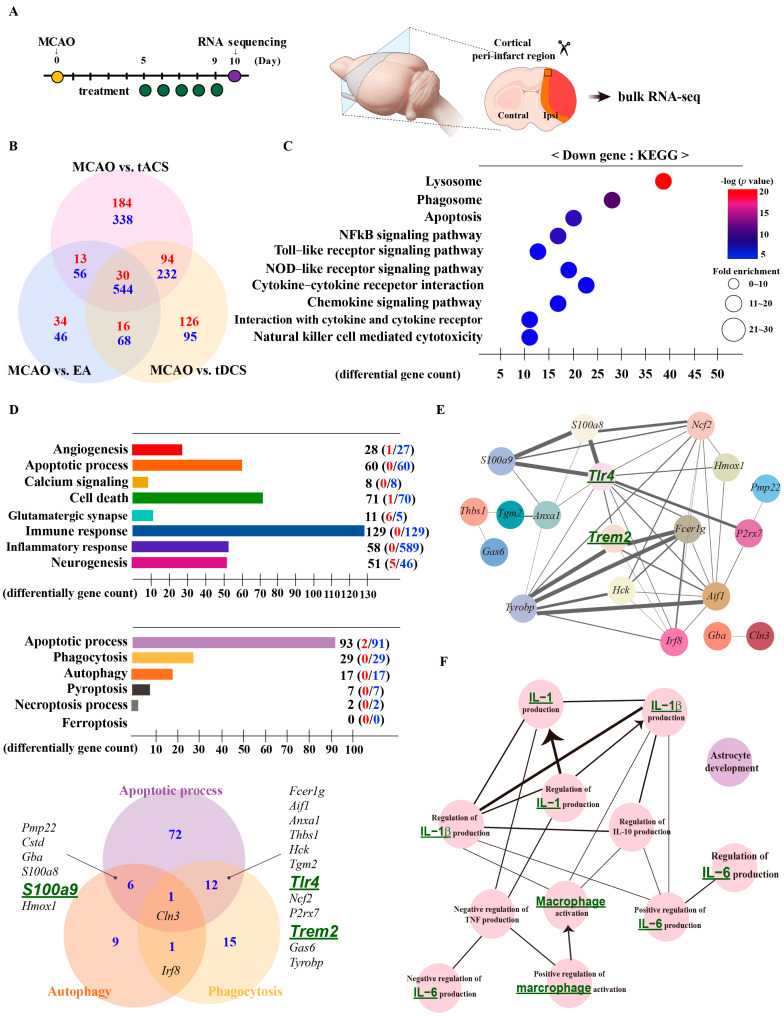
Analysis of quantitative RNA-seq to identify potent signaling pathways induced by therapeutic electrical stimulation in ischemic stroke. (**A**) Experimental schedule and schematic images of RNA-seq. (**B**) Venn diagram of DEGs in each group. Red/blue font means number of up-/down regulated genes. (**C**) Bubble plot of the top 10 KEGG pathways using 544 downregulated DEGs. Larger bubbles indicate a higher number of genes. The color of each bubble represents the *p*-value. (**D**) Upper panel: bar chart of common DEG categories, including 30 up- and 544 downregulated DEGs. Lower panel: bar chart of the cell death category using common DEGs, including 30 up- and 544 downregulated DEGs. The common genes are mainly related to apoptosis, autophagy, and phagocytosis. (**E**) Protein–protein interaction network analysis of commonly expressed genes in apoptosis, autophagy, and phagocytosis and (**F**) ClueGO analysis results of biological interactions. Each group includes three mice. RNA-seq, ribonucleic acid sequencing; DEGs, differentially expressed genes; KEGG, Kyoto Encyclopedia of Genes and Genomes.

Quantitative Ribonucleic acid sequencing (RNA-seq) was performed using samples from the cortical peri-infarct region to identify potent signaling pathways (Figure 2A). Differentially expressed genes (DEGs) compared with the MCAO-only group (|FC| > 2, *p* < 0.05) were chosen. The number of genes in each group that significantly differed from the MCAO group was visualized in a Venn diagram. We then focused on the shared electrically stimulated genes in these plots, including 30 upregulated and 544 downregulated genes (Figure 2B). The 30 upregulated DEGs were significantly enriched in 28 Gene Ontology (GO)-terms (*p* < 0.05). However, no significant differences were observed in the Kyoto Encyclopedia of Genes and Genomes (KEGG). The 59 KEGG and 525 GO-terms were significantly enriched among the 544 downregulated DEGs (*p* < 0.05). The contents of the top 10 signaling pathways identified in the KEGG analysis of the downregulated genes included lysosomes (mmu04142), phagosomes (mmu04145), and apoptosis (mmu04210) (Figure 2C). We further classified the common electrically stimulated genes into gene categories using an Excel-based differentially expressed gene analysis tool (version 4.0) based on the QuickGO (version 2.0; https://www.ebi.ac.uk/QuickGO/; accessed on 29 March 2023) database. Immune response (GO:0006955, 0 upregulated and 129 downregulated genes), apoptosis (GO:0006915, 0 upregulated and 60 downregulated genes), and cell death (GO:0048102, 1 upregulated and 70 downregulated genes) were the most potent categories activated by electrical stimulation. We focused on apoptotic processes and cell death, which were prevalent in the KEGG and category analyses, and subdivided them into the cell death pathways of ischemic stroke based on QuickGO. The DEGs were included in the order of apoptotic processes (GO:0006915, 2 upregulated and 91 downregulated genes), phagocytosis (GO:0006909, 29 downregulated genes), autophagy (GO:0006914, 17 downregulated genes), pyroptosis (GO:0070269, 7 downregulated genes), and necroptotic processes (GO:0070266, 2 downregulated genes). Most genes were involved in apoptosis, phagocytosis, and autophagy. Based on the Venn diagram, we chose 20 genes that were common to both cell death pathways (Figure 2D). Protein–protein interaction analysis to determine the relationships between the 20 genes revealed that, except for *Gba* and *Cln3*, all genes interacted using *Tlr4* and *Trem2* (PPI enrichment: 1.00 × 10^−16^) (Figure 2E). Finally, ClueGO examined the biological interactions between the 20 common genes, revealing 12 GO-terms related to biological processes. The most significant relationship was the control of IL-6 production, involving IL-1 production and macrophage activation (Figure 2F). Based on these analyses, we selected seven genes for further investigation: triggering receptor expressed on myeloid cells 2 (*Trem2*), S100 calcium-binding protein A9 (*S100a9*), galectin 3 (*Lgals3*), Toll-like receptor 4 (*Tlr4*), ionized calcium-binding adapter molecule 1 (*Iba1*), interleukin 1 (*Il-1*), and interleukin 6 (*Il-6*).

### 2.3. Effects of Therapeutic Electrical Stimulation on Inflammatory Cytokine Regulation in Ischemic Stroke

**Figure 3:**
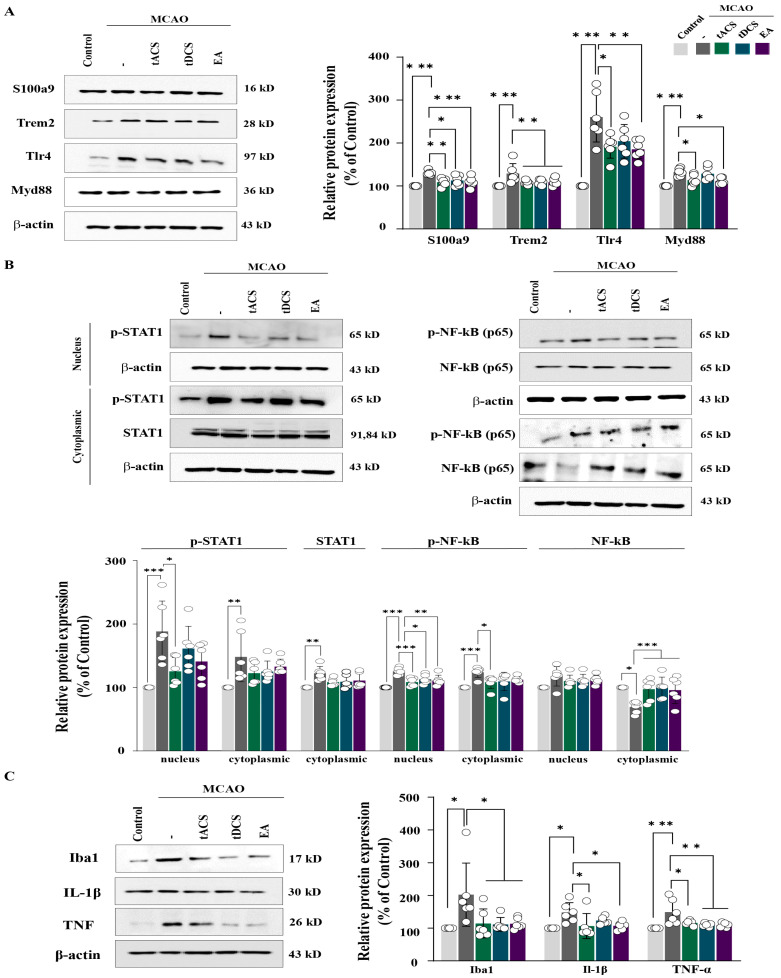
Effects of therapeutic electrical stimulation on inflammatory cytokines and related signaling pathways in ischemic stroke. (**A**) Western blot and quantification of S100a9, Trem2, Tlr4, and Myd88. (**B**) Western blot and corresponding bar graphs of p-SATA1/STAT1 and p-NF-kB/NF-kB in the nucleus and cytoplasm. (**C**) Western blot and corresponding quantification of IL-1β, Iba1, and TNF-α. These findings revealed that electrical stimulation in an ischemic stroke model decreased the levels of cytokines such as IL-1β and TNF-α by modulating STAT1 and NF-kB activation. All data are expressed as mean ± standard error of the mean (n = 6/group); * *p* < 0.05, ** *p* < 0.01, and *** *p* < 0.001 vs. each group using one-way analysis of variance with Tukey’s test. White dot means individual values. MCAO, middle cerebral artery occlusion.

Based on the results of the RNA-seq analysis, we verified 11 factors related to inflammatory cytokines (S100a9, Trem2, Lgals3, Tlr4, myeloid differentiation primary response 88 [Myd88], signal transducer and activator of transcription 1 [STAT1], nuclear factor kappa-light-chain-enhancer of activated B cells [NF-kB], Iba1, Il-6, Il-1β, and tumor necrosis factor alpha [TNF-α]) using Western blotting. Among the neuroinflammation triggers and receptors, the expression levels of S100a9 and Trem2 decreased significantly in all electrical stimulation groups compared with those of the MCAO-only group (S100a9: F_[4,25]_ = 9.689, *p* < 0.001; Trem2: F_[4,25]_ = 5.34, *p* = 0.003). Moreover, the expression levels of Tlr4 and Myd88 were significantly lower in the MCAO + tACS and MCAO + EA groups than in the MCAO group but not in the MCAO + tDCS group (Tlr4: F_[4,25]_ = 16.34, *p* < 0.001; Myd88: F_[4,25]_ = 9.551, *p* < 0.001) (Figure 3A). Regarding the transcription factors for cytokine production, p-STAT1 expression levels in the nucleus of the stimulated groups were generally decreased compared with those in the MCAO-only group but were significant only in the MCAO + tACS group (nucleus: F_[4,25]_ = 7.094, *p* < 0.001; cytoplasm: F_[4,25]_ = 4.104, *p* = 0.011). Compared with the MCAO-only group, the electrical stimulation groups had markedly lower nuclear levels of p-NF-kB (nucleus: F_[4,25]_ = 12.705, *p* < 0.001; cytoplasm: F_[4,25]_ = 5.697, *p* = 0.002; Figure 3B). IL-1β expression levels were significantly decreased in the MCAO + tACS and MCAO + EA groups, and TNF-α expression in all electrically stimulated groups (IL-1β: F_[4,25]_ = 4.297, *p* = 0.009; TNF-α: F_[4,25]_ = 4.76, *p* < 0.001). The expression of the microglia marker Iba1 was also significantly decreased in all electrically stimulated groups compared with that in the MCAO-only group (Iba1: F_[4,25]_ = 4.354, *p* = 0.008; Figure 3C). Therefore, therapeutic electrical stimulations modulated the decrease in inflammatory cytokines, including IL-1β and TNF-α, in the ischemic stroke mice model by activating the STAT1 and NF-kB signaling pathways.

### 2.4. Immunofluorescence Analysis of the Signaling Pathways of Inflammatory Cytokines in Response to Therapeutic Electrical Stimulation in Ischemic Stroke

**Figure 4:**
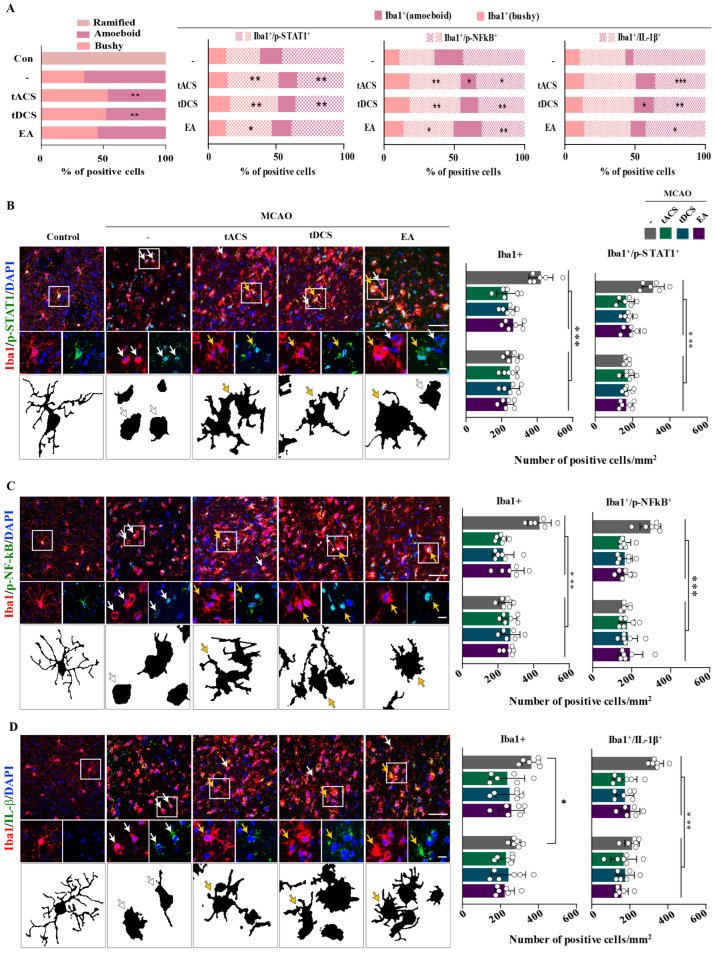
Effect of therapeutic electrical stimulation on IL-1β production and related factors in activated microglia in ischemic stroke. (**A**) Effect of electrical stimulations on microglia morphology in the ischemic stroke model. (**B**) Photomicrographs and corresponding quantification of Iba1/p-STAT1 double-positive cells in the perilesional cortex. (**C**) Photomicrographs and corresponding bar graphs of Iba1/p-NF-kB double-positive cells. (**D**) Photomicrographs and corresponding bar charts of Iba1/IL-1β double-positive cells. Activated microglia are shown in amoeboid (white arrows) and bushy (yellow arrows) forms. Amoeboid and bushy microglia were present in the MCAO surgery groups, and compared with the MCAO group, the amoeboid form decreased significantly in the MCAO + tACS and MCAO + tDCS groups. The number of Iba1/p-STAT1, Iba1/p-NF-kB, and Iba1/IL-1β double-positive cells, as well as the percentage of cells, in amoeboid microglia are significantly decreased in all electrically stimulated groups. All data are expressed as mean ± standard error of mean (n = 6/group); * *p* < 0.05, ** *p* < 0.01, and *** *p* < 0.001 vs. each group using one-way analysis of variance with Tukey’s test. Scale bars, 30 and 50 μm. White dot means individual values. MCAO, middle cerebral artery occlusion.

The resting ramified microglial form was only observed in the control group, whereas the perilesional cortices of the MCAO groups displayed bushy and amoeboid forms. The amoeboid form was significantly less expressed in the MCAO + tACS and MCAO + tDCS groups than in the MCAO-only group, whereas the bushy form was slightly more expressed; however, no significant between-group difference was observed (bushy form: F_[4,25]_ = 7.056, *p* = 0.002; amoeboid form: F_[4,25]_ = 7.056, *p* = 0.002; Figure 4A). Moreover, the number of Iba1/p-STAT1 and Iba1/p-NF-kB double-positive cells varied depending on microglial morphology. Compared with the MCAO-only group, all electrical stimulation groups demonstrated significant decreases in Iba1/p-STAT1 and Iba1/p-NF-kB double-positive amoeboid (Iba1/p-STAT1: F[3,20] = 13.678, *p* < 0.001; Iba1/p-NF-kB: F[3,20] = 15.527, *p* < 0.001) but not bushy forms (Figure 4A–C), the same was true for Iba1/IL-1β in all electrically simulated groups (F[3,20] = 4.032, *p* = 0.021; Figure 4A,D). In addition, all electric stimulation groups showed a significant decrease in the percentage of Iba1/p-STAT1, Iba1/p-NF-kB, and Iba1/IL-1 double-positive amoeboid forms (Iba1/p-STAT1: F[3,20] = 7.368, *p* = 0.002; Iba1/p-NF-kB: F[3,20] = 7.296, *p* = 0.002) and an increase in the bushy forms (Iba1/p-STAT1: F[3,20] = 6.566, *p* = 0.003; Iba1/p-NF-kB: F[3,20] = 7.987, *p* = 0.001; Iba1/IL-1: F[3,20] = 9.403, *p* < 0.001) compared to the MCAO-only group (Figure 4A). These results suggest that therapeutic electrical stimulation regulated cytokine IL-1β production by activating STAT1 and NF-kB signaling, especially in the amoeboid form of microglia.

### 2.5. Immunofluorescence Analysis of Neurotrophic Factors (NTFs) in Response to Therapeutic Electrical Stimulation in Ischemic Stroke

**Figure 5:**
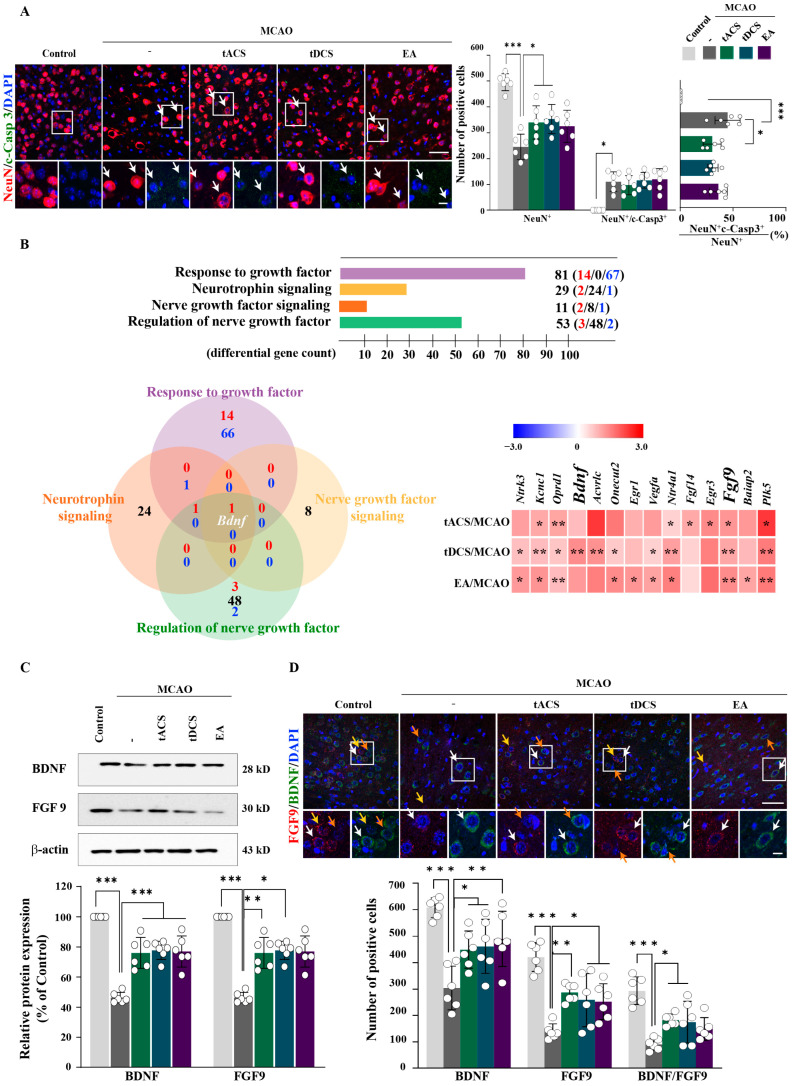
Effect of therapeutic electrical stimulation on neuronal survival and neurotrophic factors in ischemic stroke. (**A**) Photomicrographs and corresponding quantification of Iba1/c-Casp3 double-positive cells (white arrows) in the perilesional cortex. (**B**) Bar chart and Venn diagram of categories and heat map of neurotrophic factors using common DEGs of electrical stimulation. (**C**) Western blot and corresponding bar graphs of BDNF and FGF9. (**D**) Photomicrographs and corresponding quantification of BDNF- and FGF9-positive cells in the perilesional cortex (white arrows, BDNF/FGF9-, yellow arrows, FGF9-, and orange arrows, BDNF-positive cells). All electrical stimulations significantly increase the expression of neurotrophic factors, such as BDNF and FGF9, in ischemic stroke and are involved in neuronal survival. All data are expressed as mean ± standard error of the mean (n = 6/group); * *p* < 0.05, ** *p* < 0.01, and *** *p* < 0.001 vs. each group using one-way analysis of variance with Tukey’s test. Scale bars, 30 and 50 μm. Red/blue font means number of up-/down regulated genes. White dot means individual values. MCAO, middle cerebral artery occlusion.

Compared with the control group, the MCAO-only group showed a significantly decreased number of neuronal nuclei (NeuN)-positive cells in the perilesional site; however, these numbers were significantly higher in the MCAO + tACS and MCAO + tDCS groups (F_[4,25]_ = 16.763, *p* < 0.001). Regarding neuronal death, the number of NeuN/c-Casp3 double-positive cells was significantly higher in the MCAO-only group than in the control group but decreased slightly in all electrically stimulated groups; however, the percentages of these cells were significantly lower in the MCAO + tACS and MCAO + tDCS groups than in the MCAO-only group (F_[4,25]_ = 28.231, *p* < 0.001; Figure 5A). We next performed RNA-seq of NTFs to identify the factors motivating neuronal survival following electrical stimulation. Using DEGs commonly expressed during electrical stimulation, we analyzed the following categories associated with NTFs: response to growth factor (GO:0070848, 14 upregulated and 67 downregulated genes), neurotrophin signaling (GO:0038179, 2 upregulated, 24 contra-regulated, and 1 downregulated gene), nerve growth factor signaling (GO:0038180, 2 upregulated, 8 contra-regulated, and 1 downregulated gene), and regulation of nerve growth factor (GO:1990089, 3 upregulated, 48 contra-regulated, and 2 downregulated genes).

The Venn diagram and heatmap analysis revealed that, compared with the MCAO-only group, all electrically stimulated groups had significantly increased fibroblast growth factor 9 (*Fgf9*) expression; moreover, brain-derived neurotrophic factor (*Bdnf*) was common in all four categories (Figure 5B). Compared with the MCAO-only group, Western blot analysis revealed a significant increase in BDNF and FGF9 in all electrically simulated groups compared, except for FGF9 in the MCAO + EA group (BDNF: F_[4,25]_ = 42.094, *p* < 0.001; FGF9: F_[4,25]_ = 42.912, *p* < 0.001; Figure 5C). Immunohistochemistry analysis revealed that all types of electrical stimulation significantly increased the number of BDNF- and FGF-positive cells compared with MCAO-only and that the numbers of BDNF/FGF9 double-positive cells were significantly higher in the MCAO + tACS and MCAO + tDCS groups than in the MCAO-only group (BDNF: F_[4,25]_ = 10.24, *p* < 0.001; FGF9: F_[4,25]_ = 15.597, *p* < 0.001; BDNF/FGF9: F_[4,25]_ = 13.966, *p* < 0.001; Figure 5D). Therefore, therapeutic electrical stimulation improved neuronal survival in ischemic stroke by triggering NTFs such as BDNF and FGF9.

### 2.6. Effects of Peripheral Somatosensory Stimulation Using EA on Cortical Activity in Ischemic Stroke

**Figure 6:**
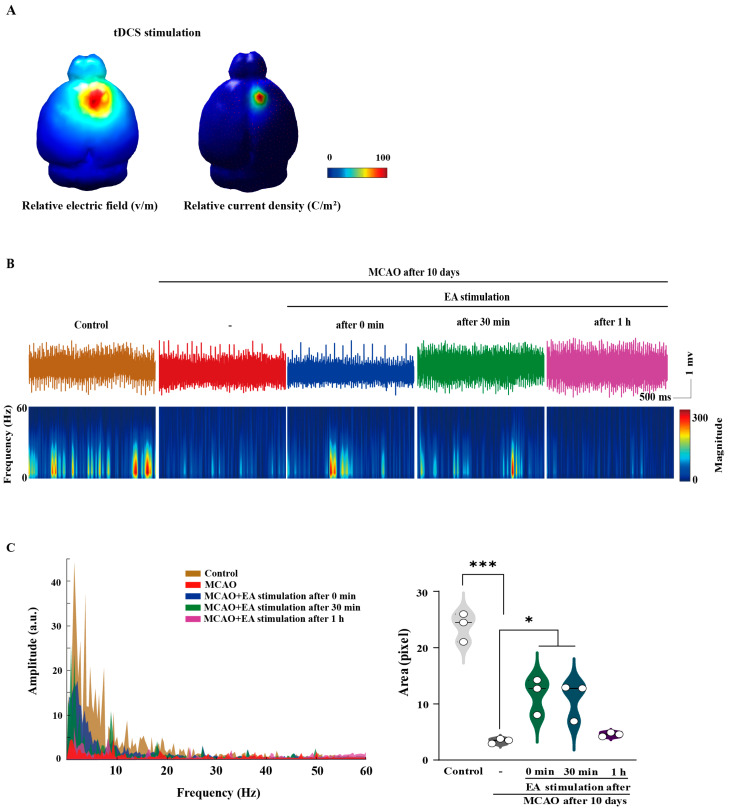
Effect of peripheral somatosensory stimulation by electroacupuncture (EA) on the EEG of the cortical in ischemic stroke. (**A**) Computational modeling of transcranial alternating- and direct current-stimulations. The predicted electrical fields are mainly observed around the targeted electrode area, with a higher relative peak electric potential and current density than other cortices. (**B**) Representative traces and spectrogram of each group. Orange: control; red: MCAO; blue: 0 min (immediately after EA stimulation; green: 30 min after EA stimulation; violet: 1 h after EA stimulation. (**C**) Line graph and area quantification of amplitude based on frequency. Brain oscillation in a lesional cortex of the ischemic model is altered by EA stimulation. EA stimulation changed brain oscillation in the ischemic stroke model. All data are expressed as mean ± standard error of the mean (n = 3/group); * *p* < 0.05, and *** *p* < 0.001 vs. each group using one-way analysis of variance with Tukey’s test. Color bar means light grey, Control; dark grey, MCAO; Green, MCAO + tACS; Blue, MCAO + tDCS; Pupple, MCAO + EA group. White dot means individual values. EEG, electroencephalogram; MCAO, middle cerebral artery occlusion.

In computational modeling, applying tDCS resulted in an increased relative peak electric potential and current density at the target area compared with other cortical areas, suggesting the regulation of cortical physiology (Figure 6A). Subsequently, we investigated whether peripheral somatosensory stimulation via EA altered cortical activity at the site of ischemic stroke damage using electroencephalogram (EEG) monitoring. At all wavelengths up to 60 Hz, the naïve control and MCAO groups demonstrated active cortical frequencies typically < 20 Hz. Moreover, the frequency of the MCAO-only group generally decreased at wavelengths < 20 Hz compared with the control group, even though the amplitude area decreased significantly. However, the EEG frequency and amplitude increased markedly immediately and 30 min after EA stimulation and decreased 1 h after stimulation (F[4,10] = 34.056, *p* < 0.001; Figure 6B,C). These findings suggested that EA stimulation via peripheral somatosensory stimulation influenced neurophysiological changes at the lesion sites in the ischemic brain.

## 3. Discussion

We investigated the benefits of therapeutic electrical stimulation in a mouse model of ischemic stroke, focusing on a molecular pathway for neuroprotection from early stroke insults. Our primary findings were as follows: (1) therapeutic electrical stimulation can improve motor dysfunction; (2) each electrical stimulation method induced the expression of different genes for stroke remission, although numerous common genes were also expressed at the subacute stage; (3) electrical stimulation regulated inflammatory cytokines, including IL-1β and TNF-α, through STAT1 and NF-kB signaling, which was the fundamental underlying mechanism; (4) the neuroprotective effects revealed an association with NTFs, particularly BDNF and FGF9; and (5) peripheral somatosensory electrical stimulation also provided a therapeutic effect by regulating cortical activity. Hence, our results suggest that therapeutic electrical stimulation may serve as an efficient adjuvant therapy for patients with stroke and may be the basis for combined therapy involving transcranial cortical- and peripheral somatosensory-stimulation.

Electrical stimulation is frequently used to aid motor recovery in patients with stroke by directly activating cortical excitability or indirectly through peripheral nerve somatosensory stimulation [5,6,7,11]. In this study, we tested several therapies, including high-definition (HD)-tACS and HD-tDCS—both of which directly stimulate transcranial cortical activity—as well as somatosensory-stimulated EA. We subsequently assessed the efficacy of these therapies through the assessment of motor function recovery in an animal model of early-stage stroke. EA did not demonstrate significant recovery in some gait analyses using the catwalk test; however, all electrical stimulations investigated in this study reduced motor dysfunction from ischemic stroke insults, demonstrating their efficacy as stroke rehabilitation therapies.

Clinical investigations have shown that the infarct volume is related to stroke severity [24]; hence, therapies to prevent neuronal death are recommended. Thus, the penumbra is a suitable conceptual target for neuroprotection [19,20]. Additionally, as different treatments work in different ways to activate therapeutic neural networks [25], we performed a transcriptomic analysis of the penumbra to identify the underlying molecular mechanisms. Surprisingly, more commonly expressed genes (30 upregulated and 544 downregulated genes) were identified in response to the three different forms of electrical stimulation, indicating a shared therapeutic mechanism. The most common type of neuronal death after stroke is apoptosis, which is caused by intrinsic or extrinsic factors. Nevertheless, other multiple cell death processes, including autophagy, phagocytosis, necroptosis, and pyroptosis, are also implicated [21]. The genes commonly expressed following the three types of therapeutic electrical stimulation included those associated with apoptosis, phagocytosis, and autophagy, the major neuronal death types in stroke pathogenesis.

Most of the targets identified in the transcriptomic analysis were brain immune-related targets in response to damage-associated molecular patterns (DAMPs), including S100a9 and its receptors (Tlr4 and Myd88), a phagocytosis receptor (Trem2), signaling cascade complexes (STAT1, NF-kB), and cytokines (IL-6, IL-1β, and TNF-α) [26,27,28,29]. DAMPs trigger the brain’s immune system through cellular sensors, including TLRs of pattern-recognition receptors (PRRs) and Trem2 of non-PRRs [29,30]. Tlr4 is frequently implicated in stroke and initiates the downstream Myd88-dependent pathway [27,30,31]. Therefore, Tlr4 activation is essential in the brain’s immune response to trigger the release of inflammatory cytokines via NF-kB. This serves as a determinant for the onset of inflammation in ischemic stroke, subsequently upregulating target genes, including IL-1β and TNF-α [26,27,30,32].

Western blot analysis verification of the selected targets in this study revealed significantly decreased expressions of S100a9, Trem2, Tlr4, Myd88, and p-NF-kB in the nucleus, and IL-1β and TNF-α in all electrically stimulated groups, except for Tlr4, Myd88, and IL-1β in the tDCS group. Additionally, p-STAT1 expression in the nucleus decreased significantly only after tACS treatment. These findings imply that all electrical stimulation methods exerted neuroprotective effects in the penumbral region by modulating inflammatory cytokines, including IL-1β and TNF-α, by regulating STAT1 and NF-kB activation, which may modulate the activity of factors involved in the brain immune system. Moreover, electrical stimulation significantly decreased the expression of Iba1, a microglial activation biomarker.

Acute inflammatory responses to ischemic injury are mostly driven by early microglial activation [33]. Microglial polarization is triggered by DAMPs and IFNγ stimulation, which activate the NF-kB and STAT1 pathways, similar to macrophages, resulting in the release of cytokines including IL-1β and TNF-α that cause neuronal death following stroke [34,35,36]. Among the inflammatory cytokines, IL-1 is a key player in the apoptotic process of acute and chronic inflammation [37,38]. The findings of the immunofluorescence analysis in the present study demonstrated that electrical stimulation significantly decreased the expression of p-NF-kB, p-STAT1, and IL-1β in the amoeboid-form microglia to prevent neuronal cell death.

The effects of the therapeutic electrical stimulations in the present study may be linked to the molecular pathways that activate NMDA receptors and trigger the production of neurotrophic growth factors [16,39,40,41]. We focused on BDNF and FGF9 in the transcriptome analysis as common high-significance growth factors because different growth factors are also expressed in commonly expressed genes through electrical stimulation. Activating NTFs, including BDNF and FGF9, allows for electrical stimulation to improve neuronal survival.

In this study, we applied electrical stimulation to an ischemic stroke model by differentiating between transcranial cortical- and peripheral somatosensory-stimulations. Our results revealed the underlying molecular mechanism by focusing on shared expressed genes. These findings imply that therapeutic cortical- and peripheral-stimulations share a common neuroprotective pathway in the penumbra region; thus, EEG analysis was conducted to identify whether peripheral somatosensory stimulation, including EA, affects cerebral cortex activity at the lesion site. Electrical stimulation, including EA stimulation via peripheral nerves, modulates neurophysiological patterns in the brain [42]. Consequently, non-invasive brain stimulation techniques, including tACS and tDCS, may also indirectly alter brain circuits by transcutaneously stimulating nearby nerves including the occipital nerve of the scalp [43,44,45], similar to EA, which involves spinal connections from acupoints to brain networks primarily through the ventrolateral funiculus [12].

Another aspect to consider of this study is how transcranial cortical- and peripheral somatosensory-stimulation affects gene expression in the brain. Evidence of responses of the central nervous system to therapeutic electrical stimulation remains lacking. Nevertheless, previous studies have suggested that the therapeutic effects of transcranial current stimulation are mediated through the regulation of NMDA receptors and Ca^2+^ channels, further linking downstream molecular cascades [16,17,46,47,48]. The somatosensory stimulation of EA facilitates physiological processes via the regulation of ionotropic glutamate receptors, including the modulation of NMDA receptors and Ca^2+^ influx, which have therapeutic effects [12,18,49]. The associated therapeutic electrical stimulation may have a therapeutic effect on genes in the brain by regulating ionotropic glutamate receptors and Ca^2+^ levels; however, further studies are required to confirm this.

The present study identified therapeutic molecular targets of electrical stimulation through transcriptomic analysis and confirmed these targets using additional biomedical techniques; however, this study has limitations. Because some other genes were commonly modulated by these methods, the analysis was restricted to the neuronal death pathway associated with the recovery of motor function in ischemic stroke; therefore, other common genes showing changes following electrical stimulation might exert neuroprotective effects through other molecular pathways.

The findings of the present study serve as a basis for molecular mechanisms to use therapeutic electrical stimulation as adjuvant therapy in patients with stroke, even in the early stages, who have few therapeutic choices available and experience substantial motor impairment following ischemic stroke. Moreover, the independent application of cortical and peripheral somatosensory stimulation, pairing tACS or tDCS with EA, may synergistically improve motor dysfunction in patients with stroke via a shared pathway that regulates neuronal cell death and further enhances the beneficial effects on motor deficits.

## 4. Materials and Methods

### 4.1. Experimental Procedures

Sixty-nine male C57BL/6J mice (6 weeks of age) were purchased from Hana Biotech (Pyeongtaek-si, Republic of Korea) and used following a two-week adaptation period. Only the male mice were used in this study because estrogen, a neuroprotective hormone, has been shown to affect infarction intensity. After randomization, the mice were divided into five groups: control, MCAO-only, MCAO + tACS (MCAO treated with tACS), MCAO + tDCS (MCAO treated with tDCS), and MCAO + EA (MCAO treated with EA). All electric stimulation was treated once daily for 5 days, starting on day 5 after MCAO surgery. The five groups underwent behavioral, Western blotting, and immunohistochemical analyses. Four groups, except the control, were used for transcriptome analysis, whereas the control and MCAO + EA groups were used for EEG analysis. The Animal Ethics Committee of the Pusan National University (Approval Number PUN-2023-0267) approved this study.

### 4.2. Animals

Hana Biotech (Pyeongtaek -si, Republic of Korea) provided 69 male C57BL/6 mice at 6 weeks of age. The sample size was determined using G*Power 3.1 software and based on the findings of a prior study on motor function after ischemic stroke (n = 5 for each group; effect size f = 0.8, α = 0.05, and β = 0.2). Thus, each group had a sample size of ≥5 (range, 5–6) mice. After randomization, the mice were divided into five groups: control, MCAO-only, MCAO + tACS, MCAO + tDCS, and MCAO + EA. Mice were randomly divided into different groups, allocated in a blinded manner.

### 4.3. MCAO Model

To establish a mouse model of ischemic stroke, we inserted a 7-0 monofilament (Doocol Corporation, Sharon, MA, USA) into the common carotid artery and advanced it through the internal carotid artery to occlude the middle cerebral artery for 40 min, after which reperfusion was performed. The PeriFlux Laser Doppler System 5000 (Perimed, Stockholm, Sweden) was attached to the mouse skull to monitor blood flow. The mice were anesthetized intraoperatively using 20% O_2_ and 80% N_2_O with 2% isoflurane (200 mL/min O_2_, 800 mL/min N_2_O; VSP Corporation, Choongwae, Seoul, Republic of Korea) administered with a calibrated vaporizer (Midmark NIP 3000, Orchard, OH, USA).

### 4.4. Electrical Stimulation

HD-tACS and HD-tDCS were delivered using ring-based electrodes with a 0.5-mm radius. The mice were anesthetized with isoflurane, and the center of the active electrode was placed over the scalp to stimulate the motor cortex (1.25 mm lateral, 1.2 mm from the bregma). HD-tACS (20 Hz, 89.1 µA/mm^2^) and anodal HD-tDCS (intensity, 55 µA/mm^2^; charge density, 66,000 C/m^2^) were applied continuously for 20 min using a constant current stimulator with eight channels (Neuro Rehab, Yeosu, Republic of Korea) for 5 consecutive days. Anodal HD-tDCS was applied to the contralesional cortex and referenced over the back as the extracephalic region. EA stimulation was delivered at acupoints corresponding to Sanyinjiao (SP6, located above the medial malleolus) and Zusanli (ST36, located lateral to the anterior margin of the tibia below the knee) in humans using two stainless-steel needles connected to an electrical stimulator (Pulsemaster Multichannel Stimulator SYS-A300, World Precision Instruments, Berlin, Germany). EA stimulation was conducted for 20 min at 2-Hertz stimulation of 1.0 mA once daily for 5 days. The control and non-stimulated groups were connected to the system without current stimulation under isoflurane anesthesia for 20 min.

### 4.5. Behavioral Assessments

We used the rotarod, wire grip, open-field, and catwalk tests to assess motor function. Motor behavior tests were run 1 and 2 days after the last electrical stimulation. All mice were handled and pre-trained before MCAO surgery (five times), and blinded observers and independent researchers conducted all behavioral tests.

Rotarod test: The average latency of each mouse falling down a rotating rod (6 cm diameter and 7.5 cm length; Panlab S.L.U., Barcelona, Spain) was measured. The speed of the rotating rod was progressively increased from 4 to 20 rpm.

Wire grip test: we measured the use of the tail and forelimbs, when hanging from a wire with a height of 45 cm, using the average score.

Open-field test: Mice were placed in an open-field box (30 × 30 × 40 cm) and free movement in the box was measured. The mice were allowed to adjust for 5 min and free movement was recorded for 20 min. After 20 min, the box was cleaned with 70% ethanol to remove scent. Data were analyzed using Smart version 2.5.18 tracking software (Panlab S.L.U., Barcelona, Spain).

Catwalk test: The mice were placed on a runway. Walking was considered successful when the mice walked on the runway without interference or hesitation. The test involved five replicate crossings for each mouse, and images of the paw prints were analyzed using CatWalk version 7.1 software (Noldus Information Technologies, Wageningen, The Netherlands).

### 4.6. RNA-Seq

For RNA-seq analysis, three mice from each group were selected based on their performance in the wire grip test following electrical stimulation after MCAO. The mice were anesthetized with sodium pentobarbital (50 mg/kg, 051100, SCI Pharmtech Inc., Taoyuan, Taiwan), and the cortical peri-infarct regions of their brains (from +0.37 to +0.97 mm anterior to the bregma) were quickly isolated for RNA-seq at Ebiogen Inc. (Seoul, Republic of Korea). Fast quality control was performed for the RNA-seq reads. QuantSeq 3′ mRNA-Seq Library Prep Kit (Lexogen, Inc., Greenland, NH, USA) was used to prepare libraries following the manufacturer’s instructions, and purified libraries were quantified using a Qubit 2.0 and Agilent 2100 Bioanalyzer. High-throughput sequencing was performed via single-end 75 sequencing using NextSeq 500 (Illumina, Inc., San Diego, CA, USA) based on a cluster of the cBot library. Trimming was performed using fxtrimmer (version 0.0.14), and the trimmed reads were mapped to the UCSC mm10 database using bowtie2 (version 2.3.5.1). We determined differentially expressed genes (DEGs) using Bedtools (version 2.27.1) (Quinlan AR, 2010) based on counts from unique and multiple alignments. Ebiogen Inc. provided an Excel-based Differentially Expressed Gene Analysis (ExDEGA) software (version 4.0) for data mining. DEGs were determined based on a significant filter of variables with a *p*-value < 0.05 and an absolute value of a log_2_-fold change > 2 in each comparison group.

### 4.7. Western Blotting

The cortical peri-infarct region was immediately separated from the lesional hemisphere. Protein (20 μg/mL) was loaded onto SDS-PAGE and transferred onto a nitrocellulose membrane (Whatman GmbH, Dassel, Germany) after constant quantification. The membrane was incubated with specific antibodies, and the bands were visualized using a chemiluminescent substrate. The images were captured using Image Quant LAS-40000 (version 1.3, GE Healthcare Life Science, Uppsala, Sweden). The antibodies used were as follows: β-actin (1:500, A2066, Sigma-Aldrich, Saint Louis, MO, USA), Galectin3 (Lgals3; 1:1000, MA1-940, Invitrogen Thermo Fisher Scientific, Carlsbad, CA, USA), triggering receptor expressed on myeloid cells 2 (Trem2; 1:200, 59621, Cell signaling, Danvers, MA, USA), Toll-like receptor 4 (Tlr4; 1:1000, 2219, Cell signaling), myeloid differentiation primary response 88 (Myd88; 1:100, PA5-19918, Invitrogen), ionized calcium-binding adapter molecule 1 (Iba1; 1:500, SC-32725, Santa Cruz Biotechnology, Santa Cruz, Dallas, TX, USA), S100a9 (1:1000, NB110-89726, Novus biologicals, Centennial, CO, USA), interleukin-1β (IL-1β; 1:1000, ab234437, Abcam, Cambridge, UK), IL-6 (1:1000, ab208113, Abcam), tumor necrosis factor-α (TNF-α; 1:1000, ab66579, Abcam), phosphorylated signal transducer and activator of transcription 1 (p-STAT1; 1:1000, 9167s, Cell signaling), STAT1 (9172, Cell signaling), phosphorylated nuclear factor kappa B (p-NF-kB; 1:1000, 3033s, Cell signaling), NF-kB (1:1000, ab16502, Abcam), brain-derived neurotrophic factor (BDNF; 1:500, ab101747, Abcam), fibroblast growth factor 9 (FGF9; 1:100, sc8413, Santa Cruz Biotechnology), and goat anti-mouse or rabbit immunoglobulin G (IgG; ADI-SAB-100, ADI-SAB-300; Enzo Life Science, Farmingdale, NY, USA).

### 4.8. Immunofluorescence

The isolated brain tissues were post-fixed in paraformaldehyde overnight and soaked in 30% sucrose for 48 h at 4 °C. Subsequently, the brain tissues were sectioned using a cryostat (CM3050, Leica Microsystems, Wetzlar, Germany) to a 20-micrometer thickness (from +0.37 to +0.97 mm anterior to bregma), including the cortical peri-infarct region for RNA-seq and Western blotting. The tissue sections were incubated with specific antibodies and mounted with a DAPI-containing mounting medium (#H-1200, Vector Laboratories, Burlingame, CA, USA). Images were captured using a fluorescence K1-fluo confocal microscope (#SMTH-0607-100T, NANOSCOPE Systems, Daejeon, Republic of Korea). Positive cells were quantified, and microglial morphology was analyzed using ImageJ software (version 1.54g) and eye counting in a blinded manner. The following antibodies were used: Iba1 (1:500, sc32725, Santa Cruz Biotechnology; 1:500, 019-19741, Wako pure chemical corporation, Osaka, Japan); p-STAT1 (1:500, 9167s, Cell signaling); p-NF-kB (1:500. 3033s, Cell signaling); IL-1β (1:1000, ab234437, Abcam); neuronal nuclei (NeuN; 1:500, MAB377, Merck Millipore, Burlington, MA, USA); cleaved caspase 3 (c-Casp3; 1:200, 9661s, Cell signaling); BDNF (1:500, ab101747, Abcam); FGF9 (1:200, sc8413, Santa Cruz Biotechnology); goat anti-mouse or rabbit IgG-Alexa Fluor^TM^ 488 (A-11001, A-11008, Invitrogen Thermo Fisher Scientific) and -Alexa Fluor^TM^ 594 (A-11005, A-11012, Invitrogen Thermo Fisher Scientific).

### 4.9. Computational Simulation

Magnetic resonance (MR) (9.4 T MRI scanner, Biospec 94/20 usr, Bruker, Germany) and micro-computed tomography (micro-CT) images (SkyScan 1278, Bruker, Germany) were acquired from the head and neck of C57BL/6 mice for three-dimensional (3D) simulation. The micro-CT images were reconstructed using the SkyScan NRecon software (version 1.7.4.6). The skull mesh model was obtained from micro-CT images, whereas the skin and brain mesh models were obtained from MR images and itk-SNAP (itk-SNAP v3.8.0; www.itksnap.org; accessed on 2 August 2023) for the 3D anatomical head. The boundary element model solver with the MoM algorithm used three mesh models (conductivity properties: scalp, 0.465; skull, 0.015; cerebrospinal fluid, 1.65; brain, 0.3) for electrical field analysis. The graphical user interface SW for simulating transcranial current stimulation using the MoM algorithm was implemented using MATLAB 2022b (MathWorks, Natick, MA, USA).

### 4.10. Electroencephalograms

The cortical activity in response to EA treatment was monitored using the Intan RHS stim/Recording system (Intan Technologies, Los Angeles, CA, USA). The mice were fixed via stereotaxic under isoflurane anesthesia. Hybrid graphene electrodes (Gbrain Corporation, Inchon, Republic of Korea) were placed over the motor area, including the damaged area, after carefully removing the skull (radius, 0.5 cm). The hybrid graphene electrodes were connected to an RHS interface board for recording. Cortical rhythms were monitored in the control and MCAO groups. Mice in the MCAO group were assessed four times: before, at 0 min (immediately), 30 min, and 1 h after EA stimulation. Cortical activity was analyzed using MATLAB 2020b (MathWorks, Natick, MA, USA) and ImageJ (version 1.54g).

### 4.11. Statistical Analyses

One-way analysis of variance and Tukey’s post hoc multiple comparison tests were used for statistical comparisons. Data were analyzed using Sigmastat (version 12.5, Systat Software, San Jose, CA, USA). Results are presented as mean ± standard error of mean, and a *p*-value < 0.05 was considered statistically significant.

## Data Availability

All data required to assess the conclusions of the manuscript are presented in the paper.

## References

[1] Feigin V.L., Nguyen G., Cercy K., Johnson C.O., Alam T., Parmar P.G., Abajobir A.A., Abate K.H., Abd-Allah F., GBD 2016 Lifetime Risk of Stroke Collaborator (2018). Global, Regional, and Country-Specific Lifetime Risks of Stroke, 1990 and 2016. N. Engl. J. Med..

[2] Campbell B.C.V., Khatri P. (2020). Stroke. Lancet.

[3] Di Pino G., Pellegrino G., Assenza G., Capone F., Ferreri F., Formica D., Ranieri F., Tombini M., Ziemann U., Rothwell J.C. (2014). Modulation of brain plasticity in stroke: A novel model for neurorehabilitation. Nat. Rev. Neurol..

[4] Jolugbo P., Ariens R.A.S. (2021). Thrombus Composition and Efficacy of Thrombolysis and Thrombectomy in Acute Ischemic Stroke. Stroke.

[5] Hummel F., Celnik P., Giraux P., Floel A., Wu W.H., Gerloff C., Cohen L.G. (2005). Effects of non-invasive cortical stimulation on skilled motor function in chronic stroke. Brain.

[6] Hermann D.M., Chopp M. (2012). Promoting brain remodelling and plasticity for stroke recovery: Therapeutic promise and potential pitfalls of clinical translation. Lancet Neurol..

[7] Peruzzotti-Jametti L., Cambiaghi M., Bacigaluppi M., Gallizioli M., Gaude E., Mari S., Sandrone S., Cursi M., Teneud L., Comi G. (2013). Safety and efficacy of transcranial direct current stimulation in acute experimental ischemic stroke. Stroke.

[8] Reinhart R.M.G., Nguyen J.A. (2019). Working memory revived in older adults by synchronizing rhythmic brain circuits. Nat. Neurosci..

[9] Bikson M., Esmaeilpour Z., Adair D., Kronberg G., Tyler W.J., Antal A., Datta A., Sabel B.A., Nitsche M.A., Loo C. (2019). Transcranial electrical stimulation nomenclature. Brain Stimul..

[10] NIH Consensus Conference (1998). Acupuncture. JAMA.

[11] Xu M., Li D., Zhang S. (2018). Acupuncture for acute stroke. Cochrane Database Syst. Rev..

[12] Zhao Z.Q. (2008). Neural mechanism underlying acupuncture analgesia. Prog. Neurobiol..

[13] Celnik P., Paik N.J., Vandermeeren Y., Dimyan M., Cohen L.G. (2009). Effects of combined peripheral nerve stimulation and brain polarization on performance of a motor sequence task after chronic stroke. Stroke.

[14] Rizzo V., Terranova C., Crupi D., Sant’angelo A., Girlanda P., Quartarone A. (2014). Increased transcranial direct current stimulation after effects during concurrent peripheral electrical nerve stimulation. Brain Stimul..

[15] Liebetanz D., Nitsche M.A., Tergau F., Paulus W. (2002). Pharmacological approach to the mechanisms of transcranial DC-stimulation-induced after-effects of human motor cortex excitability. Brain.

[16] Fritsch B., Reis J., Martinowich K., Schambra H.M., Ji Y., Cohen L.G., Lu B. (2010). Direct current stimulation promotes BDNF-dependent synaptic plasticity: Potential implications for motor learning. Neuron.

[17] Wischnewski M., Engelhardt M., Salehinejad M.A., Schutter D., Kuo M.F., Nitsche M.A. (2019). NMDA Receptor-Mediated Motor Cortex Plasticity After 20 Hz Transcranial Alternating Current Stimulation. Cereb. Cortex.

[18] Shin H.K., Lee S.W., Choi B.T. (2017). Modulation of neurogenesis via neurotrophic factors in acupuncture treatments for neurological diseases. Biochem. Pharmacol..

[19] Donnan G.A., Baron J.C., Ma H., Davis S.M. (2009). Penumbral selection of patients for trials of acute stroke therapy. Lancet Neurol..

[20] Esposito E., Li W., Mandeville E.T., Park J.H., Sencan I., Guo S., Shi J., Lan J., Lee J., Hayakawa K. (2020). Potential circadian effects on translational failure for neuroprotection. Nature.

[21] Tuo Q.Z., Zhang S.T., Lei P. (2022). Mechanisms of neuronal cell death in ischemic stroke and their therapeutic implications. Med. Res. Rev..

[22] Hordacre B., McCambridge A.B., Ridding M.C., Bradnam L.V. (2021). Can Transcranial Direct Current Stimulation Enhance Poststroke Motor Recovery? Development of a Theoretical Patient-Tailored Model. Neurology.

[23] Yarbrough C.K., Ong C.J., Beyer A.B., Lipsey K., Derdeyn C.P. (2015). Endovascular Thrombectomy for Anterior Circulation Stroke: Systematic Review and Meta-Analysis. Stroke.

[24] Yuen M.M., Prabhat A.M., Mazurek M.H., Chavva I.R., Crawford A., Cahn B.A., Beekman R., Kim J.A., Gobeske K.T., Petersen N.H. (2022). Portable, low-field magnetic resonance imaging enables highly accessible and dynamic bedside evaluation of ischemic stroke. Sci. Adv..

[25] Faingold C.L. (2008). Electrical stimulation therapies for CNS disorders and pain are mediated by competition between different neuronal networks in the brain. Med. Hypotheses.

[26] Sansing L.H., Harris T.H., Welsh F.A., Kasner S.E., Hunter C.A., Kariko K. (2011). Toll-like receptor 4 contributes to poor outcome after intracerebral hemorrhage. Ann. Neurol..

[27] Fang H., Wang P.F., Zhou Y., Wang Y.C., Yang Q.W. (2013). Toll-like receptor 4 signaling in intracerebral hemorrhage-induced inflammation and injury. J. Neuroinflamm..

[28] Liesz A., Dalpke A., Mracsko E., Antoine D.J., Roth S., Zhou W., Yang H., Na S.Y., Akhisaroglu M., Fleming T. (2015). DAMP signaling is a key pathway inducing immune modulation after brain injury. J. Neurosci..

[29] Klegeris A. (2021). Regulation of neuroimmune processes by damage- and resolution-associated molecular patterns. Neural Regen. Res..

[30] Fitzgerald K.A., Kagan J.C. (2020). Toll-like Receptors and the Control of Immunity. Cell.

[31] Urra X., Cervera A., Obach V., Climent N., Planas A.M., Chamorro A. (2009). Monocytes are major players in the prognosis and risk of infection after acute stroke. Stroke.

[32] Caso J.R., Pradillo J.M., Hurtado O., Lorenzo P., Moro M.A., Lizasoain I. (2007). Toll-like receptor 4 is involved in brain damage and inflammation after experimental stroke. Circulation.

[33] Rodhe J., Burguillos M.A., de Pablos R.M., Kavanagh E., Persson A., Englund E., Deierborg T., Venero J.L., Joseph B. (2016). Spatio-temporal activation of caspase-8 in myeloid cells upon ischemic stroke. Acta Neuropathol. Commun..

[34] Gordon S., Taylor P.R. (2005). Monocyte and macrophage heterogeneity. Nat. Rev. Immunol..

[35] Garcia-Culebras A., Duran-Laforet V., Pena-Martinez C., Ballesteros I., Pradillo J.M., Diaz-Guzman J., Lizasoain I., Moro M.A. (2018). Myeloid cells as therapeutic targets in neuroinflammation after stroke: Specific roles of neutrophils and neutrophil-platelet interactions. J. Cereb. Blood Flow. Metab..

[36] Tschoe C., Bushnell C.D., Duncan P.W., Alexander-Miller M.A., Wolfe S.Q. (2020). Neuroinflammation after Intracerebral Hemorrhage and Potential Therapeutic Targets. J. Stroke.

[37] Bis J.C., Heckbert S.R., Smith N.L., Reiner A.P., Rice K., Lumley T., Hindorff L.A., Marciante K.D., Enquobahrie D.A., Monks S.A. (2008). Variation in inflammation-related genes and risk of incident nonfatal myocardial infarction or ischemic stroke. Atherosclerosis.

[38] Endres M., Moro M.A., Nolte C.H., Dames C., Buckwalter M.S., Meisel A. (2022). Immune Pathways in Etiology, Acute Phase, and Chronic Sequelae of Ischemic Stroke. Circ. Res..

[39] Colgan L.A., Hu M., Misler J.A., Parra-Bueno P., Moran C.M., Leitges M., Yasuda R. (2018). PKCalpha integrates spatiotemporally distinct Ca^2+^ and autocrine BDNF signaling to facilitate synaptic plasticity. Nat. Neurosci..

[40] Ahn S.M., Jung D.H., Lee H.J., Pak M.E., Jung Y.J., Shin Y.I., Shin H.K., Choi B.T. (2020). Contralesional Application of Transcranial Direct Current Stimulation on Functional Improvement in Ischemic Stroke Mice. Stroke.

[41] Lee H.J., Jung D.H., Kim N.K., Shin H.K., Choi B.T. (2022). Effects of electroacupuncture on the functionality of NG2-expressing cells in perilesional brain tissue of mice following ischemic stroke. Neural Regen. Res..

[42] Chen W., Liu G., Su Y., Zhang Y., Lin Y., Jiang M., Huang H., Ren G., Yan J. (2020). EEG signal varies with different outcomes in comatose patients: A quantitative method of electroencephalography reactivity. J. Neurosci. Methods.

[43] Lefaucheur J.P., Antal A., Ayache S.S., Benninger D.H., Brunelin J., Cogiamanian F., Cotelli M., De Ridder D., Ferrucci R., Langguth B. (2017). Evidence-based guidelines on the therapeutic use of transcranial direct current stimulation (tDCS). Clin. Neurophysiol..

[44] Voroslakos M., Takeuchi Y., Brinyiczki K., Zombori T., Oliva A., Fernandez-Ruiz A., Kozak G., Kincses Z.T., Ivanyi B., Buzsaki G. (2018). Direct effects of transcranial electric stimulation on brain circuits in rats and humans. Nat. Commun..

[45] Asamoah B., Khatoun A., Mc Laughlin M. (2019). tACS motor system effects can be caused by transcutaneous stimulation of peripheral nerves. Nat. Commun..

[46] Nitsche M.A., Fricke K., Henschke U., Schlitterlau A., Liebetanz D., Lang N., Henning S., Tergau F., Paulus W. (2003). Pharmacological modulation of cortical excitability shifts induced by transcranial direct current stimulation in humans. J. Physiol..

[47] Monai H., Ohkura M., Tanaka M., Oe Y., Konno A., Hirai H., Mikoshiba K., Itohara S., Nakai J., Iwai Y. (2016). Calcium imaging reveals glial involvement in transcranial direct current stimulation-induced plasticity in mouse brain. Nat. Commun..

[48] Jung D.H., Lee J.H., Lee H.J., Park J.W., Jung Y.J., Shin H.K., Choi B.T. (2024). Therapeutic effects of a novel electrode for transcranial direct current stimulation in ischemic stroke mice. Theranostics.

[49] Liu Z., Chen X., Gao Y., Sun S., Yang L., Yang Q., Bai F., Xiong L., Wang Q. (2015). Involvement of GluR2 up-regulation in neuroprotection by electroacupuncture pretreatment via cannabinoid CB1 receptor in mice. Sci. Rep..

